# An evaluation of eye movement desensitization and reprocessing therapy delivered remotely during the Covid–19 pandemic

**DOI:** 10.1186/s12888-021-03571-x

**Published:** 2021-11-11

**Authors:** Iain W. McGowan, Naomi Fisher, Justin Havens, Simon Proudlock

**Affiliations:** 1 School of Nursing and Midwifery, Queens University, Belfast, UK; 2 Clinical Psychologist, Private Therapy Practice, Hove, UK; 3 Dr. Justin Havens Psychological Therapy, Cheltenham, UK; 4 Counselling Psychology Solutions, Stratfield Saye, Reading, UK

## Abstract

**Background:**

In addition to having a negative impact on the physical and emotional health of the population, the global Covid–19 pandemic has necessitated psychotherapists moving their practice to online environments. This service evaluation examines the efficacy of Eye Movement Desensitization and Reprocessing (EMDR) Therapy delivered via the internet.

**Methods:**

A real–world service evaluation was conducted from a self–selecting group of EMDR therapists that subscribe to either a JISCMail discussion list or either the UK or All Ireland National EMDR Associations. Author designed questionnaires were used to gather information on the efficacy of EMDR delivered online as well as client and therapist characteristics.

**Results:**

Thirty-three therapists provided efficacy data on a total of 93 patients. Statistically significant and clinically meaningful reductions were found in all four-psychometrics used both in adult and children and young people populations. Client outcome was not related to therapist experience.

**Conclusions:**

EMDR delivered via the internet can be an effective treatment for clients experiencing mental health issues.

## Background

Eye Movement Desensitization and Reprocessing therapy (EMDR) is an effective, evidence–based treatment for the treatment of several mental health issues including post–traumatic stress disorder (PTSD), depression, anxiety and eating disorders. It is recommended as a first line intervention for people experiencing symptoms associated with PTSD by the ISTSS [1] and the World Health Organization [2]. The UK, National Institute of Health and Care Excellence (NICE) [3] recommend using EMDR where trauma focused Cognitive Behavioural Therapy (CBT) is unsuccessful or the client declines CBT.

EMDR is guided by the adaptive information processing model [4] The model posits that current PTSD symptomatology is a result of maladaptive information processing of unprocessed memories stored in the brain. EMDR therapy suggests that after a traumatic experience, information processing is developed using bilateral stimulation of each brain hemisphere “resulting in new learning, elimination of emotional distress, and development of cognitive insights” [5].

The Covid–19 pandemic has had a negative impact on the mental health of the general population. In the last year, several meta–analyses have been published reporting the negative impact Covid–19 has had on the general public [6], healthcare workers [6], people living with physical health problems such as cancer [7] and those with pre–existing mental health issues [8]. This impact has been felt globally. In China, Hao et al. (2020) [8] surveyed 76 people currently in treatment for a mental health problem and 109 without. They found statistically significant differences between the group in levels of PTSD symptoms experienced, depression, anxiety and insomnia. Similar findings are reported in Saudi Arabia [9], Pakistan [10] and the Philippines [11]. In the United Kingdom, Pierce et al. [12] report a significant decline of the mental health of the general population between the start of the pandemic and April 2020. Negative impacts of Covid 19 are found in the general population in Spain [13], amongst the student population in Greece [14] and the Philippines [11], and amongst healthcare workers in Italy [6].

Most Government responses to the Covid 19 pandemic included some form of, what became colloquially known in the UK as ‘lockdown’. However, social isolation and withdrawal have long been associated with the onset of mental health problems [15]. Consequently, the potential for harm from protective policies is apparent. Tien– Huy et al. (2021) [16] conducted a global survey across 63 countries to assess the impact of quarantine/ isolation on the psychological well–being of individuals (*n* = 1871). They found a statistically significant positive association between the number of days in quarantine and increased perceived stress levels. A significant positive association was also found between perceived stress levels and being exposed to a suspected or a confirmed positive case of Covid–19 [16].

Similar findings are reported by the TMGH–Global COVID–19 Collaborative (2021) [17]. This collaborative sought to examine the presence of PTSD in individuals in insolation/ quarantine. Drawing on results from 944 responses across 57 countries, they report that people experiencing symptoms of Covid − 19 and were forced to isolate reported more PTSD than those isolating for other reasons. Being made to isolate also increased the risk of developing PTSD in comparison to voluntarily entering a period of quarantine (OR: 2.92: 95% CI: 1.84–4.74: *p* < 0.001).

It is important to recognise that, in addition to the pandemic, governments and their populations have also had to manage natural and man-made disasters that have had an impact on the mental health of individuals. For example, during 2020, the Philippines was hit by 22 tropical cyclones [18]. Rocha et al. (2021) [18] suggest that has resulted in population displacement, increased socio– economic burden on individuals and families and sudden bereavement which coupled with the pandemic may increase susceptibility to psychological distress.

Islam et al. (2021) [19] considered the psychological impact of multiple “converging systems” (p112) on the healthcare workforce. In a commentary article they argue that Yemeni healthcare workers are faced with longer working hours, no or delayed salary payments, exposure to traumatic events (either from Covid or as a result of war), an increased risk of catching Covid–19 and stigmatization. This, they contend has ed. to increased levels of PTSD, anxiety disorders, lower self-efficacy and “increased detachment from the workplace” (p113).

This has resulted in not only increased demand, but also limited access to mental health services due to mental health staff reallocation to Covid related duties, closure of out–patient services and restriction on businesses providing in person services. The initial United Kingdom Government response to the pandemic necessitated the closure of the majority of publicly funded and all private providers therapy offices requiring therapists to move their practice online.

Online psychotherapy is not new. For example, Christensen et al. [20] reported the effectiveness of the computerized MoodGym CBT programme for anxiety and depression over 15 years ago. More recently, Kuhn et al. [21] explored the effectiveness of a CBT based app designed to reduce PTSD symptoms. They report statistically significant improvements in PTSD symptoms, depressive symptoms and social functioning. NICE produced guidelines for the remote delivery of psychological therapies and recommended a number of ‘digital therapies’ for various mental health problems including depression, anxiety, substance misuse and body dysmorphic disorder [22]. Interestingly, no digital interventions for trauma specific presentations have been assessed. Additionally, these studies focus on therapy delivered through an app or webpage, where no therapist has direct involvement in the treatment.

Remote delivery of psychotherapy, where the client works with a therapist online or via the telephone [23] has been found to be both effective and acceptable to clients experiencing PTSD symptoms. Knaevelsrud & Maercker [24] compared CBT delivered via the internet against a waitlist control. They found statistically significant differences in PTSD symptomatology between the two groups after ten sessions delivered over a five-week period. Khun & Owen [21] carried out a systematic review. They found that PTSD therapy delivered remotely was as effective as in–person treatment although they note that the samples for the papers included in their reviews were primarily military veterans and male, limiting the generalization of their findings.

It is possible that online delivery of therapy could address some of the factors associated with dropout from therapy [25]. Low/ hourly paid work and travel time and cost have previously been identified as factors that drive attrition rates from therapy [26]. The flexibility afforded by online therapy may improve retention rates and, theoretically, clinical outcome [27].

### Online EMDR

EMDR therapists have been successfully treating clients remotely over the internet [28]. However, peer reviewed reports on the efficacy of EMDR delivered via the internet are scant. Lenferink Meyerbroker & Boelen [29] specifically sought papers investigating the efficacy on EMDR online. They found a single uncontrolled study [30] that used internet-based CBT with a web based EMDR tool– iEMDR. The iEMDR is reported to be based on the standard eight–phase protocol outlined by Shapiro [4] adapted for internet delivery. The web link provided in the paper (https://www.rapidtables.com/tool/EMDR.html) is inactive, thus it is not possible to verify this claim. Fifteen participants started the intervention with 11 completing. Nine participants completed the follow up questionnaires, although the time to follow up is not reported. Spence et al. [30] reported statistically significant improvements in PTSD and anxiety symptoms. The small sample size and lack of control group should counsel against generalizing their findings.

Tarquinio et al. [31] investigated the efficacy of a single EMDR session in reducing anxiety and depression levels as well as the perceived subjective level of disturbance. Seventeen nurses who were in treatment for non–Covid related issues in France had a single session of EMDR, through a video conferencing platform, using the URG–EMDR protocol [24]. They report statistically significant reductions in anxiety, depression, subjective distress, fear of returning to their workplace and fearing for their safety in relation to Covid (*p* < 0.05).

Lazzaroni et al. (2021) [32] used the EMDR protocols for Acute and Recent Traumatic events [33] with a group of adolescents and young adults currently receiving mental healthcare services (*n* = 50 (age 13– 24 years)). The participants received 3 × 1 h sessions delivered online. Lazzaroni et al. report significant improvements in anxiety levels and post–traumatic symptomatology (*p* = 0.05) post intervention.

The small sample sizes and lack of control groups in the two studies above should impress the need for caution when interpreting these findings. However, the studies provide some evidence to support the use of EMDR online.

The bilateral stimulation inherent in EMDR therapy can be delivered in several ways from asking clients to follow the therapists’ hand or wand as the therapist moves it from side to side [34]. Alternate hand taps and butterfly hugs can also be used, as can smartphones applications that deliver sounds to alternate ears [35].

We are aware of on–going RCT’s investigating the effectiveness of EMDR in an online environment [36]. This paper seeks to expand the published literature on using EMDR online.

### Aim

The aim of this service evaluation was to determine evaluate the efficacy of the online delivery of EMDR therapy. Specifically, we sought to answer the following questions;

1. Does EMDR delivered online reduce clinical symptoms of common mental health presentations?
2. Does the level of EMDR accreditation or number of years’ experience using EMDR influence the overall client outcome?

## Methods

### Recruitment of therapists & clients

EMDR therapists were recruited through the EMDR UK and Ireland JISCMail mailing list, and direct email from the EMDR UK Association (circulation 4200 therapists). The JISCMail list is an opt–in discussion forum where subscribers engage in discussion regarding EMDR. It currently has circa 1700 subscribers. Members of both the UK EMDR Association and the JISCMail mailing list are required to have undergone an approved training course and to be practicing under supervision in accordance with EMDR Europe and/or the EMDR International Association standards. We adopted a full population approach to the evaluation and as such all members of either organization were eligible to take part. No exclusion criteria were applied.

A generic mail was sent to all subscribers inviting them to take part. Interested therapists were directed to an online form that detailed the evaluation. Therapists deciding to participate provided details in respect of their level, EMDR experience, and clinical assessment tools used. Participating therapists were then asked to complete an online form giving anonymized information of client’s presentation and outcome. As the authors had no direct contact with the clients, therapists were instructed to ensure that clients had given informed consent for their anonymized data to be used. The EMDR UK Association provided support for a random draw of participating therapists where the prize was one of three £20 gift vouchers. Suggested text regarding consent for therapists to include in their treatment plans/ consent forms was provided. Recruitment of therapists and data collection took place from May 2020–Dec 2020. Google Forms was used to collect data.

### Ethics

Using the algorithm provided by the UK National Health Research Authority and UK Policy Framework for Health and Social Care Research definition of research [37] this project was not classified as research but as a service evaluation [38]. As such formal IRB ethical approval was not required [38]. All participants were provided with an electronic information sheet that detailed the background and the aims of the study as well as the requirements of taking part. Respondents gave written informed consent to take part in the evaluation, and all processes undertaken in the evaluation were carried out in accordance with relevant guidelines and policies.

### Data collection and analysis

Author designed surveys were used to collect the data. Data collected from the therapists is reported below and participating therapists then provided anonymized data on their clients including their gender, age, primary psychiatric reason for referral, pre– and post–treatment scores and the outcome measures used in practice. Given the heterogeneity of the assessment and outcomes tools used by EMDR therapists, and this project’s status as an evaluation of existing practice we did not put any restriction on the measures used by the therapist. We asked participating therapists to provide overall pre and post treatment scores in relation to the Impact of Events Scale– Revised (IES–R) [39], the General Anxiety Disorder 7 scale (GAD–7) [40], the Public Health Questionnaire 9 scale (PHQ–9) [41] and the PTSD Checklist (PCL–5) [42] and any other assessment scales they normally use for each of their clients.. Thesescales are widely used in PTSD research and clinical practice and have been shown to have good reliability and validity. Twenty five of the 33 therapists used one of the four scales above.

Of the eight that did not use any of these scales, the Subjective Units of Distress and Validity of Cognition scales [4] used routinely in EMDR therapy were reported as outcomes by one of the therapists. Other scales used by therapists were the HADS (*n* = 1) [43], the CORE (*n* = 1) [44], GHQ (*n* = 1) [45], BDI (*n*–1) [46], BAI (*n* = 1) [47], AUDIT (*n* = 1) [48], DES–II (*n* = 1) [49], the Worry about Sexual Outcomes scale (*n* = 1) [50], ITQ (*n* = 2) [51], the Moral Disengagement Scale (*n* = 1) [52], the Work and Social Adjustment Scale (*n* = 2) [53], the Driving Cognitions Questionnaire (*n* = 1) [54], and the CRIES 13 (*n* = 1) [55]. Recognizing the very small numbers using these different assessment tools we have not reported these in this paper.

Descriptive statistics are reported for both therapists and clients. The Shapiro–Wilk test for normality returned non–significant statistics suggesting normal distribution of data. Accordingly, Student t–test was used to identify significant differences between pre– and post–treatment scores. Statistical significance was set at 0.05 throughout. Minimal clinically important differences were calculated using the distribution method [56] where a difference of half the standard deviation is recognised as being clinically important. In order to address the second research question, a third dataset was constructed combining the therapist and client details to allow for cross tabulation of outcome and anonymized therapist details. The Pearson r correlation statistic was used to explore relationships between the length of time the therapist had been practicing EMDR and each of the clinical outcomes. Analysis of variance (ANOVA) was used to ascertain differences between level of accreditation and clinical outcome. A dichotomous variable of aged 18 or over and under the age of 18 was created. ANOVA was used to explore any differences in outcome by age of the client. JASP software was used to conduct the statistical analysis.

### Findings

Thirty-three therapists provided data on a total of 93 different clients. The therapists’ mean length of time since training in EMDR was 8.5 years (sd: 4.8 yrs.: range (0.5–17.5 yrs). Eight of the therapists were trained to Consultant Level, 10 were accredited EMDR therapists and the 12 had completed initial EMDR training and were working towards accreditation.

Of the 93 clients for whom data was provided, 62 (66%) identified as female, 30 (33%) as male and 1 as non–binary (1%). Ages ranged from 10 years to 72 (mean 35.5 sd 15.6 years). Thirteen (14%) of the clients were under the age of 18 years.

Psychological trauma (simple and complex) was the most common reason for seeking help, followed by anxiety and depression. Table 1 shows the reasons for referral. One of the 93 participants sought help for Covid related problems.

**Table 1:** Frequencies for ‘What was the clients’ main presenting problem?’

	Frequency	Percentage
Complex PTSD	37	39.8
Simple PTSD	19	20.4
Anxiety	14	15.1
Depression	12	12.9
OCD	3	3.2
Grief	2	2.2
Health anxiety	1	1.1
“Negative cognitions resulting from a number of traumatic events led to DSH, anxiety and depression”	1	1.1
Phobia	1	1.1
Functional neurological disorder	1	1.1
Wanting to explore negative cognitions / “behavioural stuckness”	1	1.1
Psychosis	1	1.1

Statistically significant and clinically important reductions in the reported client mean scores of the IES(R), the GAD–7, the PHQ–9 and the PCL–5 checklist was found (Table 2). Large effect sizes post–treatment was also found. Minimal clinically important difference thresholds were set at 9.10 (IES(R)), 2.61 (GAD–7), 3.64 (PHQ–9) and 6.64 (PCL–5). No significant relationship was found between length of time trained in EMDR and any of the clinical outcomes, nor was there any significant difference in the association between level of accreditation and clinical outcome. No significant differences were found in any of the four outcomes between genders or between those aged under 18 years and clients aged 18 and over.

**Table 2:** Pre–post EMDR scores, ^a^ denotes clinically important difference

	Pre (sd)		Post (sd)		Student t test statistic	Effect size Cohens ***d***	p
	n	Mean (sd)	n	Mean (sd)			
**IES(R)**	40	56.13 (24.15)	35	18.03 (19.1)a	8.66	1.46	< 0.001
**GAD–7**	42	13.95 (4.29)	41	5.32 (4.31)a	11.33	1.77	< 0.001
**PHQ–9**	44	15.14 (6.24)	43	7.28 (4.92)a	8.31	1.28	< 0.001
**PCL–5**	15	41.53 (22.21)	15	13.27 (10.94)a	6.53	1.69	< 0.001

## Discussion

This appears to be the first evaluation to report the efficacy of using the standard eight phase EMDR protocol delivered through an online medium. Using real world data, we have shown that EMDR can reduce symptomatology regardless of the age, gender or clinical presentation of the client. The findings also show that length of time practicing EMDR and level of accreditation in EMDR are not associated with outcome, suggesting that EMDR can be used successfully regardless of experience after EMDR training.

Notwithstanding the limitations outlined below, the large effect sizes found are encouraging. A recent systematic review [57] reported a small to moderate pooled effect size (Hedges g = 0.33) [57] across 23 studies using EMDR delivered in the same room as the client for the treatment of PTSD, and a large pooled effect size across 10 studies for the treatment of anxiety disorders (Hedges g = 1.07) [57].

We are also encouraged by the apparent similarities in efficacy reported by therapists working with children and young people and those working with the adult population. This is in keeping with studies that report EMDR as an effective treatment for children and young people (CYP) that have experienced a traumatic event and that clinical improvement using EMDR is independent of demographic variables such as age [58]. For example, in a meta– analysis comparing trauma based approaches to treating PTSD in CYP Khan et al. [58] reported EMDR to be more effective than CBT in reducing PTSD symptoms (SDM (95% CI) = − 0.43 (− 0.73 – –0.12), *p* = 0.006) and anxiety symptoms (SDM (95% CI) = − 0.71 (− 1.21 – –0.21), *p* = 0.005). Lazzaroni et al. (2021) [32] and Jeon et al. (2017) [59] have shown that age is not correlated with either reductions in PTSD symptoms [32] or Post Traumatic growth [59].

Our findings allude to EMDR being as effective when delivered remotely as face to face, in line with the findings of Kuhn & Owen [21] We also note that the effect sizes reported here are in excess of those reported in meta–analyses of the CBT interventions (0.66 < g < 0.83) delivered via the internet when compared to passive controls (no treatment or wait list control) [60]. Interestingly, they also found that internet CBT was superior to active control groups [60].

As with other psychotherapies, remote delivery of EMDR has significant potential benefits to clients including the reduction in travel time to and from appointments and loss of salary to attend appointments [25] as well as a reduction in stigma associated with mental health treatment [61]. Clients have control over their environment and smartphone apps that deliver clicks via earphones alternately give the client more control of the session [23]. Other applications such as bilateralstimulation.io also provide online platforms for the delivery of the bilateral stimulations used in EMDR. Additionally, internet delivered interventions have been found to be a cost–effective way to deliver psychological interventions [39].

Conversely, when considering working with clients remotely therapists need to be cognisant of potential distractions such as children, deliveries and phone calls during the sessions [23] [27]. Fisher (2021) [23] also acknowledges that the home environment may not always be a safe environment for the client to undergo EMDR therapy and therapists need to be cognisant of this when considering online work with clients.

Having access to the internet is, obviously, a pre–requisite for online therapy. Cleofas et al. (2021) [62] surveyed 952 college students and report that computer ownership and access to the internet are associated with lower levels of Covid–19 related anxieties. Rubin (2021) [63] argues that internet access should now be a social determinant of health. She notes that internet access increases with income. Subsequently, therapists need to be cognisant of the stigma associated with poverty [64] and the potential for those living in poverty to portray a more positive outlook than their current situation [65] when considering moving therapy online.

### Limitations

This evaluation has several limitations. There is a potential for ‘gatekeeper bias’, where there is the possibility that therapists providing data may only have submitted data for clients that showed improvement. We also recognise that despite high initial interest, a relatively small number of therapists provided client data. This may suggest a self–selecting group with a bias toward online EMDR. The lack of a control group and small sample size of both therapists and clients also precludes us making any generalised claims. Although we note above that client outcome appears to be independent of level of experience as an EMDR therapist, we did not collect data on previous experience of online therapy undertaken by therapist prior to the pandemic. Subsequently, we cannot comment on therapist experience working online and the impact that that may have on clinical outcome. As noted above, we are aware of several on–going Randomised Controlled Trials exploring the effectiveness of EMDR in an online environment that may address these limitations.

## Conclusion

This evaluation appears to be the first paper to report the efficacy of EMDR delivered online using real world practice data. Our findings show that a reduction in clinical symptoms can be achieved using EMDR online, however recognising the limitations of this evaluation we would urge caution in interpreting the findings. Clinical trials examining the clinical and cost effectiveness of online EMDR are required.

The Covid 19 pandemic required EMDR therapists to adopt creative and flexible responses to help meet the needs of the clients [18]. Office closures and travel restrictions to and from work meant that therapists had to move their work to online. The findings of this evaluation suggest that they did so successfully.

## Data Availability

The datasets used and/or analysed during the current study available from the corresponding author on reasonable request.
